# Soybean adaption to high‐latitude regions is associated with natural variations of *GmFT2b*, an ortholog of *FLOWERING LOCUS T*

**DOI:** 10.1111/pce.13695

**Published:** 2020-01-25

**Authors:** Li Chen, Yupeng Cai, Mengnan Qu, Liwei Wang, Hongbo Sun, Bingjun Jiang, Tingting Wu, Luping Liu, Shi Sun, Cunxiang Wu, Weiwei Yao, Shan Yuan, Tianfu Han, Wensheng Hou

**Affiliations:** ^1^ National Center for Transgenic Research in Plants Institute of Crop Sciences, Chinese Academy of Agricultural Sciences Beijing China; ^2^ Ministry of Agriculture Key Laboratory of Soybean Biology (Beijing) Institute of Crop Sciences, Chinese Academy of Agricultural Sciences Beijing China

**Keywords:** flowering promoter, gene haplotype, *Glycine max* (L.) Merr., *GmFT2b*, photoperiod, Soybean

## Abstract

Day length has an important influence on flowering and growth habit in many plant species. In crops such as soybean, photoperiod sensitivity determines the geographical range over which a given cultivar can grow and flower. The soybean genome contains ~10 genes homologous to *FT*, a central regulator of flowering from *Arabidopsis thaliana*. However, the precise roles of these soybean *FTs* are not clearly. Here we show that one such gene, *GmFT2b*, promotes flowering under long‐days (LDs). Overexpression of *GmFT2b* upregulates expression of flowering‐related genes which are important in regulating flowering time. We propose a ‘weight’ model for soybean flowering under short‐day (SD) and LD conditions. Furthermore, we examine *GmFT2b* sequences in 195 soybean cultivars, as well as flowering phenotypes, geographical distributions and maturity groups. We found that Hap3, a major *GmFT2b* haplotype, is associated with significantly earlier flowering at higher latitudes. We anticipate our assay to provide important resources for the genetic improvement of soybean, including new germplasm for soybean breeding, and also increase our understanding of functional diversity in the soybean *FT* gene family.

## Abstract 2

In an effort to understand the molecular genetic basis of flowering time in soybean, we have isolated and characterized *GmFT2b*, a soybean FT gene homolog. We used overexpression and CRISPR/Cas9‐induced targeted gene mutations to show that *GmFT2b* regulates flowering in soybean, but only under long days. We also sequenced this gene from 195 soybean cultivars in different maturity groups (MGs). Our analysis showed that one *GmFT2b* haplotype, Hap3, is found only in early‐maturing cultivars in MGs 0–2, and correlates with their ability to flower in environments located at high latitudes in northeastern China.

## 1 INTRODUCTION

Flowering time is an important trait that regulates plant adaptability and yield. Plants integrate flowering signals from a range of different internal and external cues to transition from vegetative growth to flowering to set seeds. In *Arabidopsis*, *FLOWERING LOCUS T* (*FT*) has emerged as a key integrator of multiple flowering pathways, and the FT protein is now widely accepted as being the proposed flowering hormone florigen itself or, alternatively, as the major component of a more complex florigen signal (Turck, Fornara, & Coupland, 2008; Zeevaart, 2006; Zeevaart, 2008).

The molecular mechanisms of *FT* signalling have been elucidated primarily in *Arabidopsis* (Abe et al., 2005; Corbesier et al., 2007; Jaeger & Wigge, 2007; Mathieu, Warthmann, Küttner, & Schmid, 2007), and the prevailing model of FT action has been supported by studies in rice, pumpkin, *Populus* and several other plant species (Böhlenius et al., 2006; Kojima et al., 2002; Lin et al., 2007; Tamaki, Matsuo, Wong, Yokoi, & Shimamoto, 2007; Nishikawa et al., 2007; Varkonyi‐Gasic et al., 2013; Li, Li, et al., 2013). The expression of *FT* in leaves is regulated by *CONSTANS*, a zinc‐finger transcription factor, in *Arabidopsis* (Abe et al., 2005; Wigge et al., 2005); the FT protein is produced in the leaves and transported in the phloem to the shoot apical meristem, where it acts to initiate flowering (Corbesier et al., 2007; Jaeger & Wigge, 2007). It has been suggested that FT and the bZIP‐type transcription factor FD may play a promotive role in regulating the expression of *SUPPRESSOR OF OVEREXPRESSION OF CONSTANS1* (*SOC1*) and *FRUITFUL* (*FUL*), and as a result, the FT/FD complex also upregulates the expression of floral meristem identity genes such as *APETALA1* (*AP1*) and *LEAFY* (*LFY*) in *Arabidopsis* (Benlloch et al., 2011; Kaufmann et al., 2010; Li, Zhou, et al., 2013; Wagner & Meyerowitz, 2010; Weigel, Alvarez, Smyth, Yanofsky, & Meyerowitz, 1992). Overexpression of *Hd3a*, the rice ortholog of *FT*, promotes flowering in *Arabidopsis* under short‐day (SD) conditions (Kojima et al., 2002; Lin et al., 2007; Tamaki et al., 2007). *FT* orthologs from numerous plant species representing both dicots and monocots, such as rice, tomato, potato, longan, poplar, pea, cotton, and alfalfa, have been expressed in heterologous species and have been shown to induce flowering (Guo et al., 2015; Hecht et al., 2011; Hsu et al., 2011; Kong et al., 2010; Laurie et al., 2011; Lifschitz et al., 2006; Navarro et al., 2011; Sun et al., 2011; Winterhagen, Tiyayon, Samach, Hegele, & Wünsche, 2013).

Soybean, *Glycine max* (L.) Merr., is a typical short‐day plant that is sensitive to seasonal changes in day length. Day length has an important influence on soybean flowering and growth habit, and responses to photoperiod sensitivity limit the geographical ranges of soybean cultivars. At present, at least four major flowering pathways, including the vernalization, autonomous, gibberellin (GA) and photoperiod pathways are known to regulate the floral transition process (Cai et al., 2019). At least ten *FT* gene homologs have been identified in soybean (Kong et al., 2010). These *FT* genes are the result of tandem and whole genome duplications in soybean (Thakare, Kumudini, & Dinkins, 2011; Wang et al., 2015). Among these genes, *GmFT2a* and *GmFT5a* have been found to promote flowering following the expression of the phytochrome PHYA in soybean, and show the same relationship as *AtFT* and *AtTSF* (*TWIN SISTER of FT*) in *Arabidopsis* (Nan et al., 2014; Takeshima et al., 2016; Yamaguchi, Kobayashi, Goto, Abe, & Araki, 2005). Expression of both *GmFT2a* and *GmFT5a* is up‐regulated under SD conditions, whereas they are significantly down‐regulated under long‐day (LD) conditions (Kong et al., 2010; Sun et al., 2011; Xu et al., 2013). In a previous study, we used the CRISPR/Cas9 (Clustered regularly interspaced short palindromic repeat/CRISPR‐associated 9) genome editing system to specifically induce targeted mutations in *GmFT2a* in the soybean cultivar ‘Jack.’ Homozygous *ft2a* mutant plants exhibited the late flowering phenotype under both LD and SD conditions (Cai et al., 2018). *GmFT4* is known to act as a floral repressor, and its function is similar to that of *TFL1* (*TERMINAL FLOWER 1*) in *Arabidopsis* (Zhai et al., 2014). Transgenic overexpression of *GmFT1a* delayed flowering and maturation in soybean, confirming that *GmFT1a* also functions as a flowering inhibitor (FI; Liu et al., 2018). The results suggest that FT proteins have undergone functional divergence in soybean. Currently, the results related to the homologous *FT* genes (*GmFT2a*, *GmFT5a*, *GmFT1a* and *GmFT4*) are not sufficient to explain the complex flowering mechanism in soybean. Further studies of other *FT* genes are urgently needed to better understand the functions of the 10 *FT* genes in the soybean genome.

In this study, we isolated a soybean *FT* homolog, *GmFT2b*, that is highly homologous to *GmFT2a*. We obtained transgenic overexpressing lines and also produced lines carrying mutations in *GmFT2b* using CRISPR/Cas9. Overexpression of *GmFT2b* promoted flowering under LD conditions, and *ft2b* mutants showed delayed flowering only under LD conditions. The flowering times of the *GmFT2b*‐ox and the *ft2b* mutant plants were almost the same as for WT plants under SD conditions. By analysing the expression patterns of flowering‐related genes in the *GmFT2b*‐ox and *ft2b* mutant plants, we inferred that only the flowering‐related genes in which expression was up‐ or down‐regulated sufficiently under LD conditions can regulate the flowering time. Haplotype and phenotypic analysis of *GmFT2b* indicated that Hap3 is mainly present in cultivars in MGs 0–2, which show earlier flowering times. The results of our study contribute important genetic information and provide tools for the genetic improvement of soybean such as new germplasm for soybean breeding, especially in high‐latitude regions.

## 2 MATERIALS AND METHODS

### 2.1 Plant materials and growth conditions

The soybean cultivars ‘Zigongdongdou’ (‘ZGDD’) and ‘Jack’ were used in the present study. The cultivar ‘ZGDD’ was used for gene cloning. The cultivar ‘Jack’ was used for *Agrobacterium‐*mediated transformation. Soybean seeds were germinated and the seedlings grown in a controlled culture room at 28°C under LD (16 hr light/8 hr dark) and SD (12 hr light/12 hr dark) conditions.

### 2.2 *GmFT2b* cDNA cloning

Total RNA was extracted using Trizol reagent from the trifoliolate leaves of soybean cv. ‘ZGDD’ seedlings. First‐strand cDNA was synthesized with Superscript II reverse transcriptase (TransGen Biotech, Beijing, China) and used as a template for further *GmFT2b* cDNA cloning. Amplification was performed via PCR using KOD‐plus‐Neo DNA polymerase (Toyobo, Tokyo, Japan). The sequences of the primers used for amplifying the full‐length *GmFT2b* cDNA are given in Table S1.

### 2.3 Subcellular localization of *GmFT2b*

The open reading frame (ORF) of *GmFT2b* was fused with the 5′ end of the *GFP* gene sequence in a construct under control of the CaMV 35S promoter. The *GmFT2b* gene was cloned into the p16318 plasmid (Liu et al., 2018). The recombinant fusion plasmids were introduced into onion epidermal cells by particle bombardment using a CaMV 35S:eGFP vector as control. Transformation was achieved with a PDS 1000/He device (BioRad, Hercules, CA, USA), with a 6 cm shot distance, 25 mmHg vacuum and 1,100 psi rupture disc pressure. Green fluorescent protein (GFP) fluorescence was monitored using a Zeiss LSM710 confocal microscope (Carl Zeiss, OKO, Germany).

### 2.4 Gene expression analysis

Quantitative RT‐PCR (qRT‐PCR) was performed using an ABI QuantStudio™ 7 flex Real‐Time PCR System (Applied Biosystems). To examine the expression of flowering‐related genes in the leaf and shoot apex, these tissues were sampled at 15 days after emergence (DAE) under SD conditions and 30 DAE under LD conditions. Three biological replicates were analysed, with technical replicates for each of the three biological samples. The relative expression levels were analysed using the 2^−*ΔΔCt*^ method (Livak & Schmittgen, 2001). The gene IDs and primers used to amplify each gene and the internal reference are listed in Table S1. Statistical analyses were performed using Microsoft Excel. The two asterisks represent significant differences at *p* < .01, The one asterisk represents significant differences at *p* < .05.

### 2.5 Construction of *GmFT2b*‐ox and *GmFT2b*‐CRISPR plasmids

For the overexpression construct (*GmFT2b*‐ox), the CDS of *GmFT2b* was inserted into the PTF101 vector containing a CaMV 35S promoter and a *bar* gene (Paz, Martinez, Kalvig, Fonger, & Wang, 2006). For construction of the CRISPR/Cas9 expression vector, a 20‐bp sgRNA sequence was designed using the web tool CRISPR‐P (http://cbi.hzau.edu.cn/crispr/), and its expression was driven by the *Arabidopsis U6* gene promoter. The *Cas9* sequence was inserted downstream of the CaMV 2X 35S promoter. The *bar* gene driven by a CaMV 35S promoter was used as a screening marker. A pair of DNA oligonucleotides for the sgRNA were synthesized by TSINGKE (Beijing) and annealed to generate a double‐stranded sgRNA, which was subsequently inserted into the CRISPR/Cas9 expression vector (Cai et al., 2019).

### 2.6 Soybean transformation and mutant detection

The overexpression vector (*GmFT2b*‐ox) and CRISPR/Cas9 expression vector (*GmFT2b*‐CRISPR) plasmids were transformed into *Agrobacterium tumefaciens* strains EHA101 and EHA105 via electroporation, respectively. The soybean cultivar ‘Jack’ was used for tissue culture and transformation according to a previously‐published protocol (Chen et al., 2018).

The *GmFT2b*‐overexpressing transgenic plants were screened by PCR and LibertyLink strip detection. The LibertyLink strips were used to determine the presence of the PAT protein in the transgenic plants. The potential *GmFT2b* mutants were then screened by DNA sequencing analysis. Briefly, genomic DNA was extracted from the leaves of each individual plant in the *T*_0_ generation, and the regions spanning the target sites were amplified by PCR using Phanta® Super Fidelity DNA Polymerase (Vazyme Biotech) and sequenced. Different types of gene editing events can be identified by DNA sequencing. Short base insertions or deletions (not in multiples of three) induced by CRISPR/Cas9 can lead to translational frameshift mutations. DNA from plants that were heterozygous for the mutations showed overlapping peaks on the sequencing chromatograms from the target sites to the end of the DNA fragment. The wild‐type and homozygous mutations had no overlapping peaks at the target sites. The homozygous mutant types were identified by sequence alignment against the wild‐type gene sequence (Cai et al., 2019).

### 2.7 Flowering time measurements and statistical analyses

The flowering time of each soybean plant was recorded as days from emergence to the R1 stage (the time at which the first flower appears at any node on the main stem; Fehr & Caviness, 1977). For quantitative analyses of flowering time, individual soybean plants were analysed for each genotype. Statistical analyses were performed using Microsoft Excel, and the data was analysed by ANOVA. The two asterisks represent significant differences at *p* < .01.

## 3 RESULTS

### 3.1 Cloning of *GmFT2b* and subcellular localization of the protein

The *GmFT2b* gene was isolated from ‘ZGDD’, a photoperiod‐sensitive soybean cultivar. *GmFT2b* contains a 531‐bp ORF that encodes a predicted protein of 176 amino acids. The genomic sequence of *GmFT2b* spans 3,014 bp and contains three introns and four exons. *GmFT2b* (Glyma.16g151000) is located on soybean chromosome 16 and is close to *GmFT2a* (Glyma.16g150700). *GmFT2b* shares 90.91% amino acid sequence identity with its paralog *GmFT2a* (Figure S1).

To determine the subcellular localization of the GmFT2b protein *in planta*, we fused the *GmFT2b* coding region with the gene for green fluorescent protein (GFP) under the control of the constitutive cauliflower mosaic virus (CaMV) 35S promoter and transfected the resulting p35S::GmFT2b‐GFP plasmid into onion epidermal cells. Confocal microscopy showed that the GmFT2b protein is expressed in the cytoplasm and nucleus, with a stronger signal in the nucleus (Figure S2), similar to the cellular localization of the other functional phosphatidylethanolamine binding proteins in soybean (Wang et al., 2015).

### 3.2 Overexpression of *GmFT2b* in soybean promotes flowering under LD conditions

**Figure 1:**
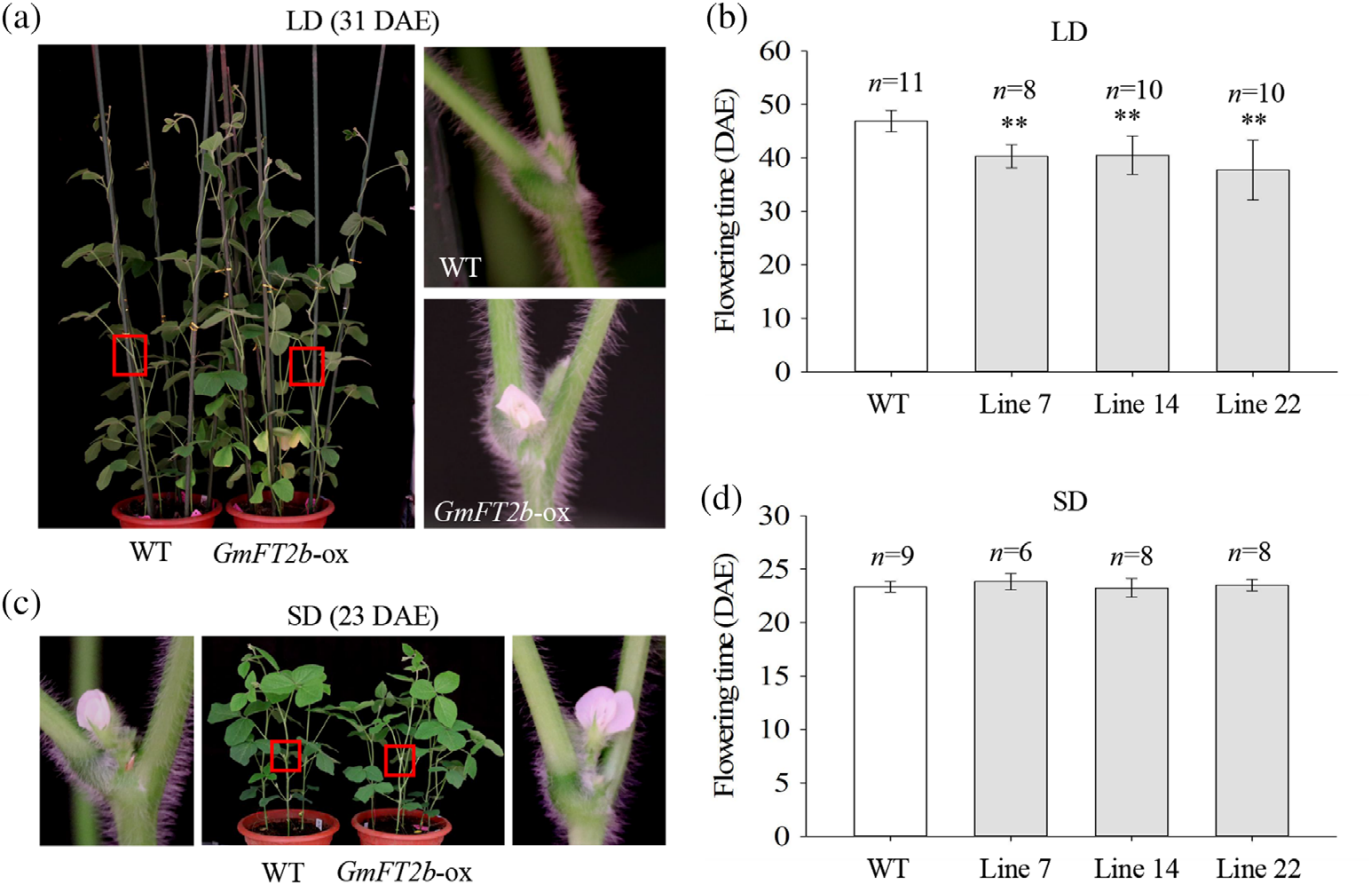
Overexpression of *GmFT2b* in soybean promotes flowering under LD conditions. (a) and (c) Phenotypes of wild‐type plants (WT) and the *GmFT2b*‐overexpressing transgenic lines under LD and SD conditions, respectively. (b) and (d) Flowering time of WT and the GmFT2b‐overexpressing transgenic lines under LDs and SDs, respectively. *n*, exact numbers of individual plants identified. **, the *GmFT2b*‐overexpressing transgenic lines exhibit highly significant early flowering (*p* < .01). DAE, days after emergence; SD, short‐day (12 hr light/12 hr dark); LD, long‐day (16 hr light/8 hr dark)

To investigate the function of *GmFT2b*, a construct containing the *GmFT2b* CDS driven by the CaMV 35S promoter was transformed into the mid‐maturing group 2 soybean cv. ‘Jack’. The positive transgenic soybean plants were detected by PCR and LibertyLink strips (Figure S3). PCR was used to check for the presence of the *bar* gene, and the LibertyLink strips were used to determine the presence of the PAT protein in transgenic plants. Under LD conditions, the WT plants flowered at 46.9 DAE, the *GmFT2b*‐overexpressing transgenic lines flowered at 40.3 (line 7), 40.5 (line 14) and 37.8 (line 22) DAE (Figure 1a, b). The flowering dates of the three transgenic lines were 6.6, 6.4 and 9.1 d earlier than the WT plants under LD conditions. Under SD conditions, the WT plants flowered at 23.3 DAE, and the three transgenic lines flowered at 23.8, 23.2 and 23.5 DAE (Figure 1c, d). The results of these LD and SD experiments demonstrate that the overexpression of *GmFT2b* can significantly promote soybean flowering under LDs, but not SDs.

### 3.3 CRISPR/Cas9‐mediated targeted mutagenesis of *GmFT2b* delays flowering time under LD conditions

**Figure 2:**
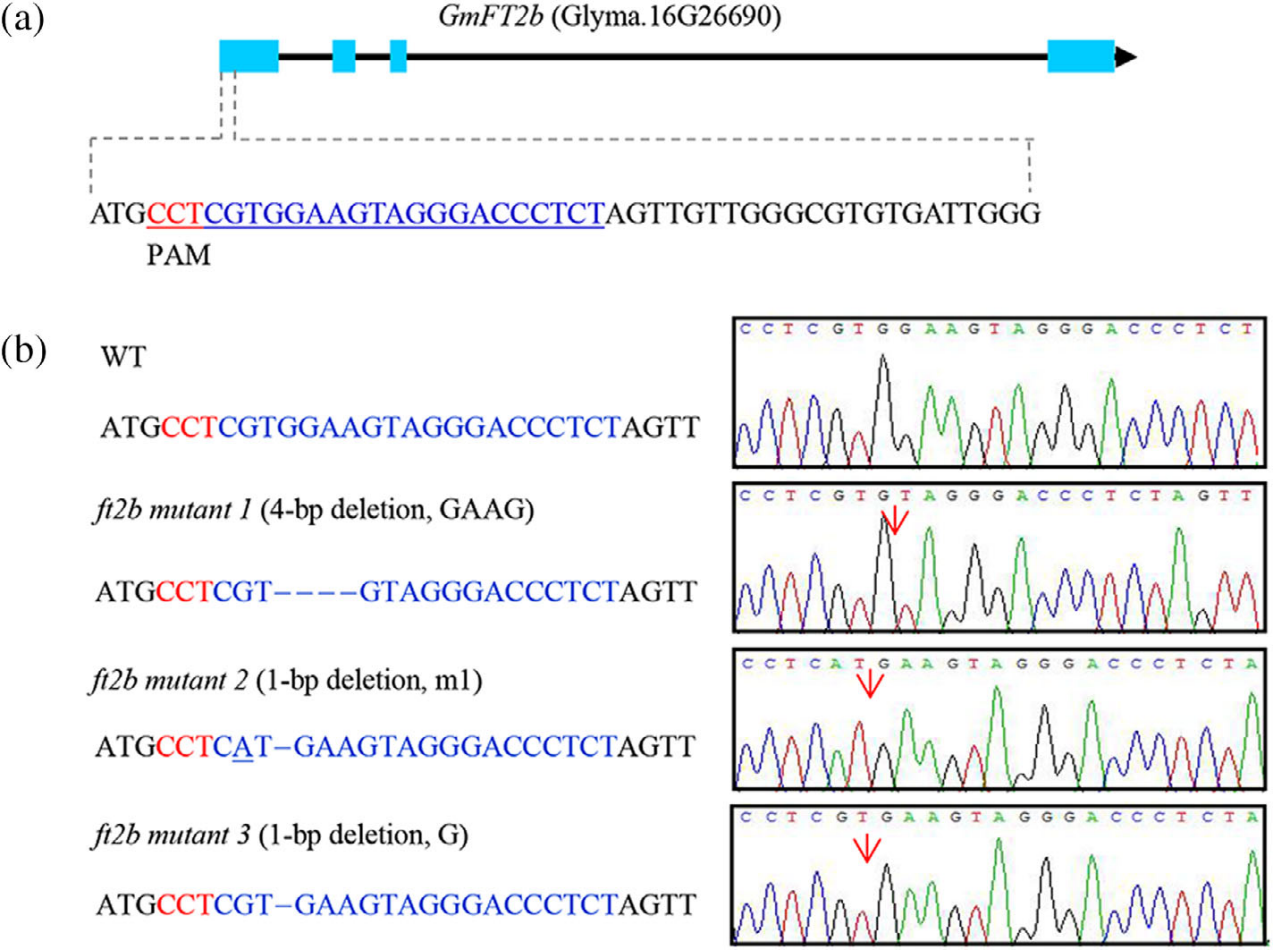
Homozygous targeted mutagenesis of *GmFT2b* induced by CRISPR/Cas9. (a) Nucleotide sequences of the target sites. (b) Sequences and sequencing chromatograms of the wild‐type and CRISPR/Cas9‐induced mutations. The red arrowheads indicate the locations of the mutations

The target site for mutagenesis was chosen in the first exon of *GmFT2b* (Figure 2a). Three types of homozygous mutations for null alleles of *GmFT2b* induced by CRISPR/Cas9 were obtained in the *T*_1_ generation (a 1‐bp deletion; a 4‐bp deletion; a 1‐bp deletion with a 1‐bp mismatch; Figure 2b). All three types of frame‐shift mutations induced by CRISPR/Cas9 at the target site in *GmFT2b* were predicted to generate premature translation termination codons.

**Figure 3:**
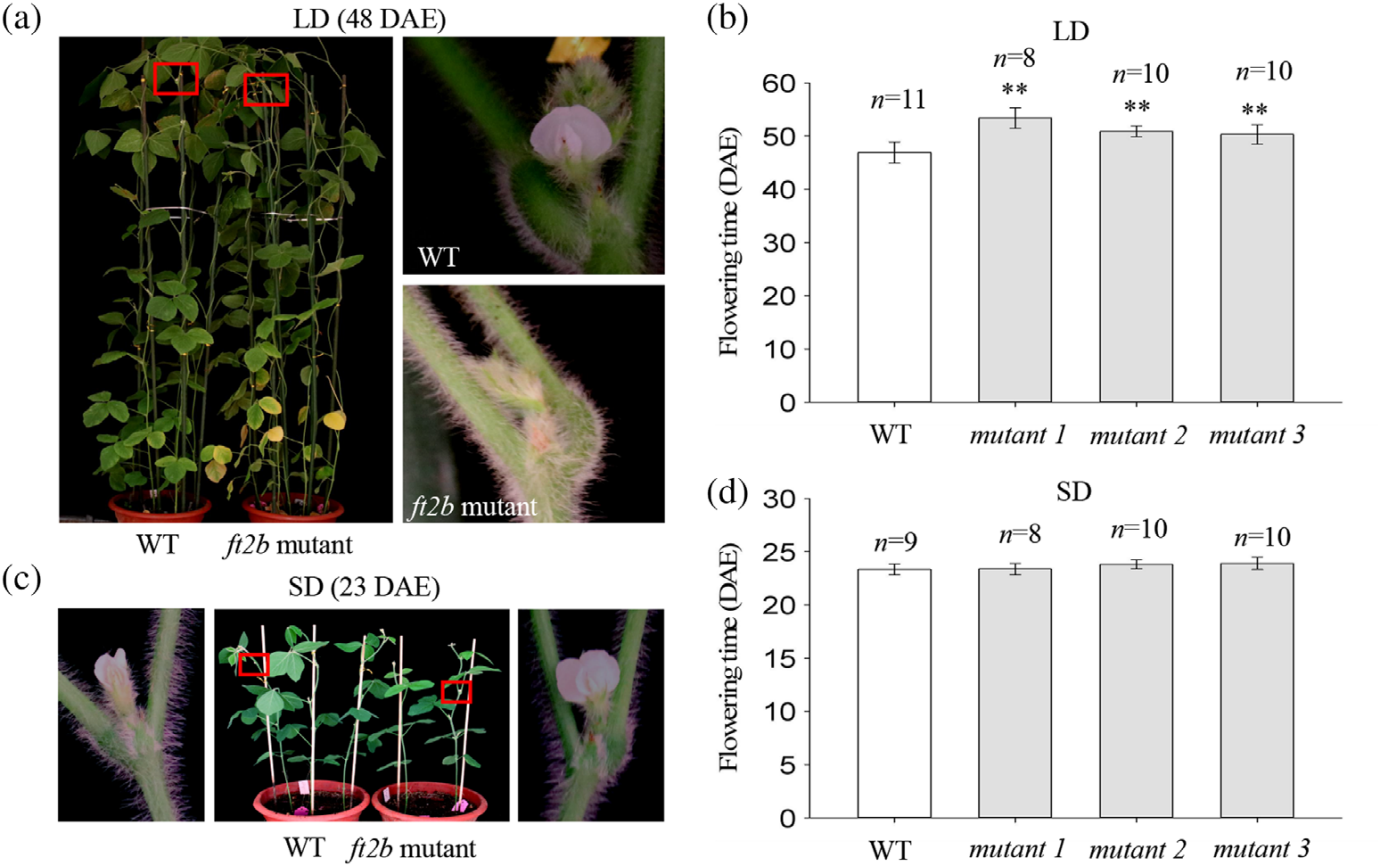
*ft2b* mutants show delayed flowering under LD conditions. (a) and (c) Phenotypes of WT and *ft2b* mutant plants under LD and SD conditions, respectively. (b) and (d) Flowering times of WT and *ft2b* mutant plants under LD and SD conditions, respectively. *n*, exact numbers of individual plants identified. **, *ft2b* mutant plants exhibit highly significant late flowering (*p* < .01). DAE, days after emergence

Under LD conditions, the *ft2b* mutants flowered at 53.4, 50.9 and 50.3 DAE, while the WT plants flowered at 46.9 DAE. The flowering dates of the three mutants were 6.5, 4.0 and 3.4 d later than the WT plants (Figure 3a, b). Under SD conditions, the *ft2b* mutants flowered at 23.4, 23.8 and 23.9 DAE, while the WT flowered at 23.3 DAE (Figure 3c,d). The results of these LD and SD experiments demonstrate that null mutations of *GmFT2b* can significantly delay soybean flowering under LDs, but not under SDs.

### 3.4 Expression of *GmFT* genes and flowering‐related genes under SD and LD conditions

Fourteen *FT* homologs and other flowering‐related genes have been isolated and characterized under SD and LD conditions in soybean; these include Gm*FT* genes (*GmFT1a*, *GmFT2a*, *GmFT2b*, *GmFT3a*, *GmFT4* and *GmFT5a*), *GmAP* genes (*GmAP1a*, *GmAP1b*,and *GmAP1c*), *GmSOC* genes (*GmSOC1a* and *GmSOC1b*), *GmFUL* (*GmFULa*), *GmLFY* (*GmLFY2*) and *GmAG*.

**Figure 4:**
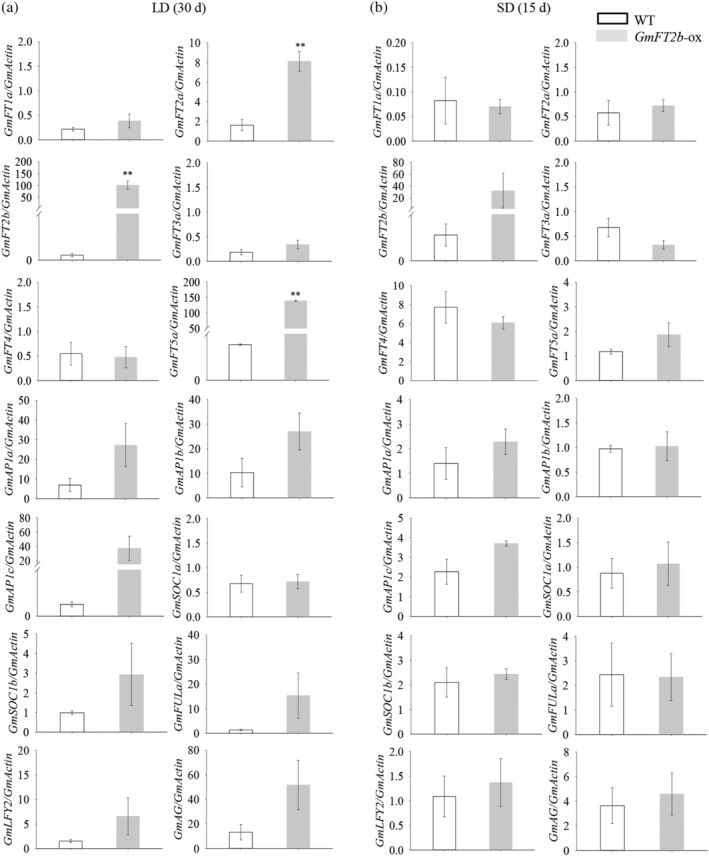
Expression patterns of *GmFT* genes and flowering‐related genes in WT plants and *GmFT2b*‐overexpressing transgenic lines under LD and SD conditions. RNA was extracted from trifoliate leaves and the shoot apex from plants grown under LD and SD conditions at 30 and 15 DAE, respectively. Relative transcript levels were quantified by qRT‐PCR and normalized to the expression of *GmActin*. Average values ± SE (standard error) for three replications are shown for each data point. **, the *GmFT2b*‐ox transgenic lines exhibit highly significant early flowering (*p* < .01)

**Figure 5:**
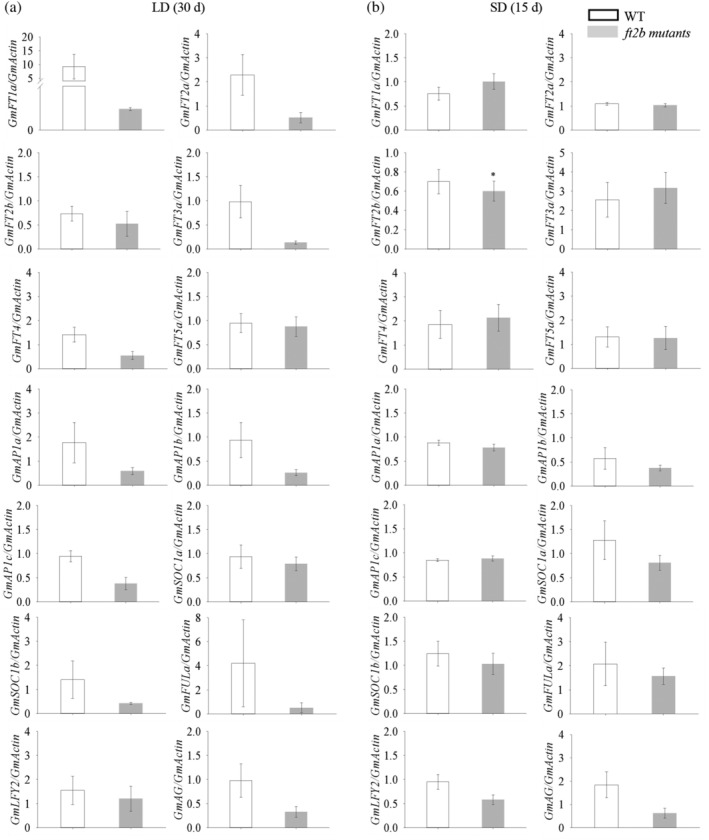
Expression patterns of *GmFT* genes and flowering‐related genes in WT plants and *ft2b* mutants under LD and SD conditions. RNA was extracted from trifoliate leaves and the shoot apex at 30 (LD) and 15 DAE (SD). Relative transcript levels were quantified by qRT‐PCR and normalized to *GmActin* expression. Average values ± SE (standard error) for three replications are shown for each data point. *, the *ft2b* mutants exhibit highly significant early flowering (*p* < .05)

In the *GmFT2b*‐ox plants, we found that the expression of *GmFT* genes including *GmFT2a* and *GmFT5a* were significantly upregulated in leaves, *GmFT4* was unchanged under LD conditions. The expression levels of *GmAP1* (*a*, *b* and *c*), *GmSOC1* (*a* and *b*), *GmFULa*, *GmLFY2* and *GmAG* were slight upregulated in the shoot apex under LD conditions. Under SD conditions, there was no significant change in the expression of *GmFT2a*, *GmFT5a*, *GmFT1a*, *GmFT3a* and *GmFT4* in leaves. The expression levels of *GmAP1* (*a* and *c*), *GmSOC1* (*a* and *b*), *GmLFY2* and *GmAG* were not affected (Figure 4). In *ft2b* CRISPR mutants, the expression levels of *GmFT1a*, *GmFT2a*, *GmFT3a*, *GmFT4*, *GmFT5a*, *GmAP1* (*a, b* and *c*), *GmSOC1* (*a* and *b*), *GmFULa*, *GmLFY2* and *GmAG* showed no marked change under LD and SD conditions (Figure 5).

### 3.5 Analysis of *GmFT2b* haplotypes revealed their distribution in soybean varieties in the different maturity groups

**Figure 6:**
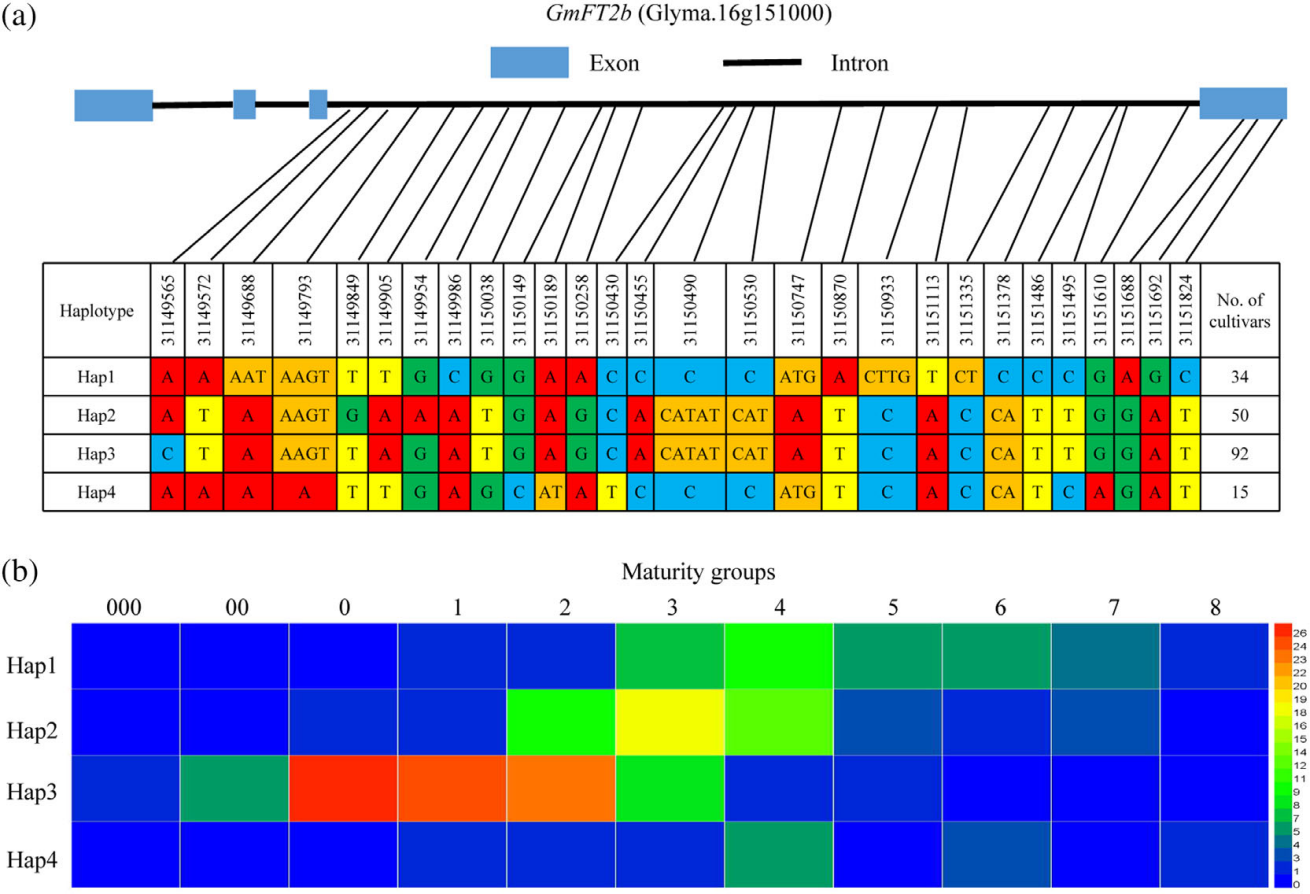
Analysis *of GmFT2b* haplotype and maturity groups for soybean varieties carrying the major haplotypes. (a) *GmFT2b* haplotypes*. GmFT2b* sequences from 195 soybean accessions were compared with those of Williams 82. Four major haplotypes with higher frequencies were identified. Site numbering and physical positions are also based on the reference genome sequence of Williams 82. The nucleotides are highlighted in different colors. The number of cultivars carrying each haplotype are listed in the right column. (b) Maturity groups of the soybean accessions with the major *GmFT2b* haplotypes. The numbers next to the colored bar indicate the number of soybean accessions with the corresponding *GmFT2b* haplotype

We investigated the nucleotide polymorphisms in the coding region of *GmFT2b* in 195 soybean accessions with varied flowering times using whole‐genome resequencing. We found single‐base changes at three sites in the fourth exon of *GmFT2b*. No predicted frameshift mutations or premature stop codons were found in the coding regions. The base changes in the fourth exon did not change the amino acid sequence and protein. The four major haplotypes with higher frequencies were identified and chosen for analysis (Figure 6a). The haplotypes of the *GmFT2b* promoter were also for analysis (Figure S4). Four haplotypes were identified in *GmFT2b* promoter, which mainly corresponded to the four haplotypes in the coding region of *GmFT2b*. We further investigated the maturity groups of the soybean varieties that carry the major *GmFT2b* haplotypes. Hap3 was mainly distributed in the varieties belonging to MGs 0, 1 and 2. Hap2 was mainly distributed in varieties in MG 2–MG 4, and Hap1 was mainly found in varieties in MGs 3 and 4. Hap4 was found to be dispersed across the various maturity groups (Figure 6b).

### 3.6 Distribution of major *GmFT2b* haplotypes in soybean varieties from diverse geographical origins

**Figure 7:**
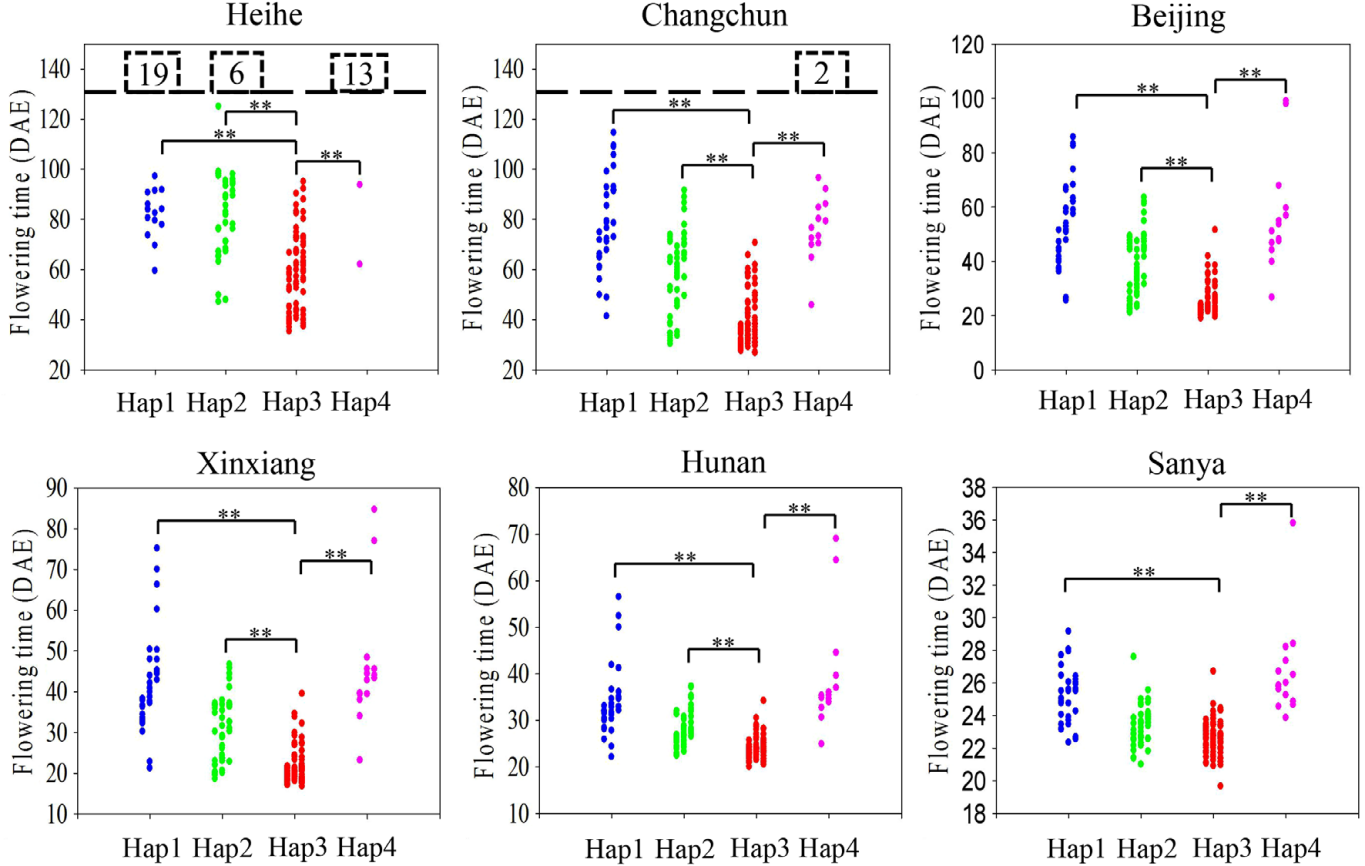
Flowering times of soybean accessions carrying the major *GmFT2b* haplotypes grown at six different latitudes. Each dot represents a soybean variety. DAE, days after emergence. The dotted line represents a flowering time of 130 DAE. The numbers in the dotted boxes indicate the number of soybean varieties that did not flower after 130 DAE. **, the Hap3 varieties exhibit highly significant early flowering times (*p* < .01)

We compared flowering times in the six environments (Heihe, Changchun, Beijing, Xinxiang, Hunan, Sanya) for varieties carrying different *GmFT2b* haplotypes (Figure 7). The flowering times were examined in regions at six different latitudes in China; from north to south these are Heihe (N50°15′, E127°27′), Changchun (N43°49′, E125°21′), Beijing (N40°09′, E116°14′), Xinxiang (N35°18′, E113°55′), Hunan (N27°49′, E112°56′) and Sanya (N18°21′, E109°10′). We found that the Hap3 varieties showed earlier flowering times, while Hap1 and Hap4 varieties flowered later. A majority of the varieties carrying *GmFT2b* haplotypes Hap1 and Hap4 were unable to flower in Heihe due to the high latitude (N50°).

We also analysed the geographical distribution of varieties with major *GmFT2b* haplotypes. Hap1 was present in a comparatively wide distribution in the southern and middle region of China. Hap2 was mostly found at higher latitude regions in the central and northern parts of the country. Hap3 occurred at higher latitudes in the northern regions. The geographical distribution of Hap4 was in the southern parts of China at low latitudes (Figure S5). Notably, all of the varieties with Hap3 can flower in the northern latitudes, which suggests that the Hap3 genotype may contribute to early flowering at high latitudes.

## 4 DISCUSSION

Soybean is a diploid species that evolved from an ancient tetraploid, and its genome has undergone homologous chromosomal recombination and reassortment of the entire genome during its long evolutionary history (Wang et al., 2015). At present, it has been shown that the soybean genome encodes at least ten members of the *GmFT* gene family. Several studies have demonstrated that *GmFT* orthologs have different temporal and spatial expression patterns as well as different functions (Endo et al., 2005; Kotoda et al., 2010). Further studies of the *GmFT2b* and other *GmFT* genes may reveal novel molecular mechanisms that act to regulate flowering in soybean.

We found that *GmFT2b*‐ox plants exhibited an early‐flowering phenotype under non‐inductive conditions (LD), while *ft2b* mutants exhibited a later flowering phenotype than did WT plants under LD conditions. Surprisingly, the flowering times of *GmFT2b*‐ox and *ft2b* mutant plants were almost the same as WT plants under SD conditions. We examined the expression patterns of some *GmFT* homolog genes and several flowering‐related genes that respond to the overexpression of *GmFT2b* in *Gmft2b*‐ox and *ft2b* mutant plants in leaves and the shoot apex. We found that overexpression of *GmFT2b* upregulated the expression of *GmFT2a* and *GmFT5a* under LD conditions in the *GmFT2b*‐ox plants, the expression of three *GmAP1* orthologs (*GmAP1a, GmAP1b, GmAP1c*), *GmSOC1b*, *GmFULa*, *GmLFY2* and *GmAG* were slight upregulated. The gene expression levels have no marked changes induced by SD. We inferred that early flowering may require higher expression levels of flowering‐related genes. In *ft2b* mutants, flowering time was delayed only under LD conditions, and the expression of almost the tested genes was slight lower than in the WT under LD conditions. Under SDs, except *GmFT2b*, the expression of other genes showed no marked change. Taken together, our results suggest that *GmFT2b* can influence the expression of *GmFT* genes and other flowering‐related genes under LD conditions in soybean. The differences in the expression levels between LDs and SDs leads to the different flowering phenotypes. Only the flowering‐related genes in which expression is up‐ or downregulated to a sufficient degree under LDs can regulate flowering time.

**Figure 8:**
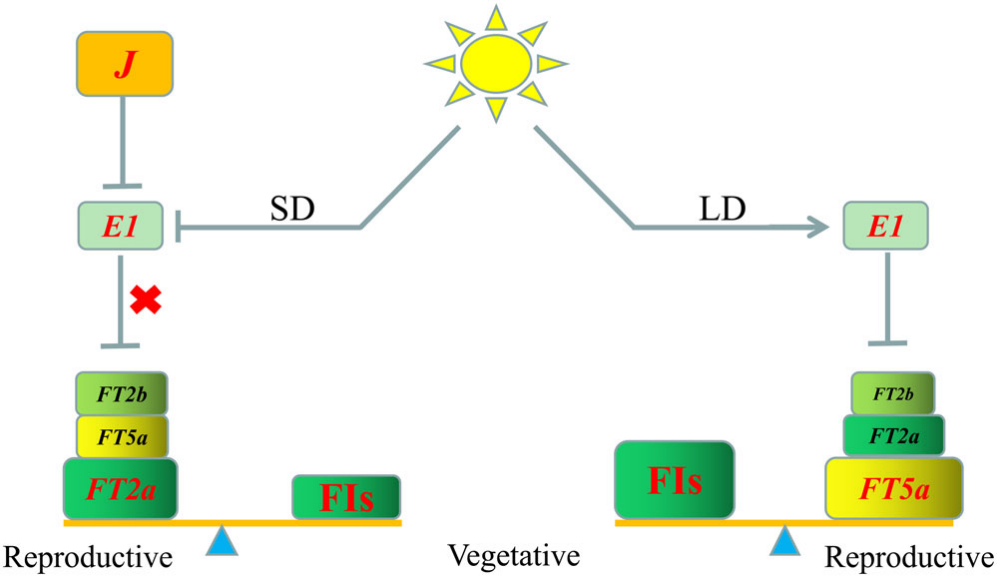
A model for the soybean flowering transition under SD and LD conditions. Gray arrows represent stimulation of gene expression. Gray T‐shaped symbols represent inhibition of gene expression. The red ‘×’ symbols represent the release of inhibition. The ‘*J*’ gene is shown in an orange box to inhibit *E1* under SD conditions. *FT2a*, *FT2b* and *FT5a* are in boxes of different colors and sizes, the box size represents the relative importance of the gene (larger is more important). FIs, flowering inhibitors; SD, short‐day (12 hr light/12 h dark); LD, long‐day (16 hr light/8 h dark)

Based on the results of the present study and previous reports (Liu et al., 2018;Lu et al., 2017; Yue et al., 2017), we propose a ‘weight’ model for soybean flowering under SD and LD conditions (Figure 8). In this model, we consider that the transformation from vegetative growth to reproductive growth is due to the balance of flowering activators and FIs. The FIs in SDs may be less than in LDs. Under SD conditions, the inhibition of *E1* on the expression of *GmFT* genes is relieved by the *J* gene (Lu et al., 2017; Yue et al., 2017). Overexpression of *GmFT2a* promotes early flowering under SD conditions, but overexpression of *GmFT5a* or *GmFT2b* does not change flowering time (Cai et al., 2019). The floral activator *GmFT2a* is more important than *GmFT5a* and *GmFT2b* under SD conditions. *GmFT2a* is sufficient to overcome the effect of FIs to promote the transformation to reproductive growth. The effects of *GmFT5a* and *GmFT2b* are less obvious. Under LD conditions, the *FT* genes are inhibited by *E1*. More flowering activators are required to overcome the increased number of FIs. All of the *GmFT2a‐*ox, *GmFT2b‐*ox, or *GmFT5a‐*ox plants will flower under LD conditions (Cai et al., 2019). The *ft5a* mutants showed much later flowering compared to the *ft2a* and *ft2b* mutants (Cai et al., 2019). Of these, *GmFT5a* is more important than *GmFT2a* and *GmFT2b* under LD conditions.

By investigating the nucleotide polymorphisms in the *GmFT2b* coding region in 195 soybean accessions, we found that the *GmFT2b* haplotypes are associated with flowering time. Soybean varieties carrying *GmFT2b* haplotype Hap3 exhibited significantly early flowering in all six environments. Most varieties with the Hap1or Hap4 haplotypes were unable to flower normally when they were grown at Heihe (higher latitude). In addition, the geographical distribution and MG distribution of the *GmFT2b* haplotypes showed that Hap3 is only found in varieties grown in higher latitude regions in northeast China that belong to earlier maturing varieties from MGs 0–2. Hap4 is found in varieties grown in lower latitude regions in southern China that belong to later maturing varieties in MGs 000–8. Our previous studies showed *GmFT2a* also has four major haplotypes with higher frequencies (Cai et al., 2019). FT2a‐Hap1 was mostly found in the Huanghuaihai. FT2a‐Hap2 was mostly found in higher latitude region in the north and the Huanghuaihai. FT2a‐Hap3 was present in the south. FT2a‐Hap4 was comparatively wide, but was rare in the northeast. FT2a‐Hap2 was mainly distributed in the varieties of MG 1, MG 2 and MG 3. The FT2a‐Hap1, FT2a‐Hap3 and FT2a‐Hap4 genotypes were not found among the earlier maturing varieties (MG 000, MG 00 and MG 0). These results suggest that different *GmFT2a* and *GmFT2b* haplotypes have considerable effects on the diversity of flowering time in soybean at different latitudes.

## CONFLICT OF INTEREST

The authors declare that they have no conflict of interest.

## AUTHOR CONTRIBUTIONS

L.C., Y.C. and M.Q. performed the experiments. L.C. and W.H. wrote the manuscript. L. W, T.W. and L.L. provided the data for the loci in the 195 soybean accessions of the diversity panel. W.Y. assisted in soybean transformation. S.S. and C.W. provided soybean varieties. S.Y. and B.J. participated in some experiments. W.H. and T.H. designed and advised on the experiments and revised the manuscript.

## Supporting information


**Appendix** **S1**: Supporting Information
