# Supplementary material for: Soybean adaption to high‐latitude regions is associated with natural variations of GmFT2b, an ortholog of FLOWERING LOCUS T
Source: Plant Cell Environ. 2020 Jan 25;43(4):934–44. doi: 10.1111/pce.13695 (PMC7154755; doi:10.1111/pce.13695)
Supplement: Supplementary file 1 — Appendix S1: Supporting Information [file PCE-43-934-s001.pdf]

Supporting Information

Article title: Soybean adaption to high-latitude regions is associated with natural variations of *GmFT2b*, an ortholog of *FLOWERING LOCUS T*

Authors: Li Chen[1,2+], Yupeng Cai[1,2+], Mengnan Qu[2+], Liwei Wang[2], Hongbo Sun[2], Bingjun Jiang[2], Tingting Wu[2], Luping Liu[2], Shi Sun[2], Cunxiang Wu[2], Weiwei Yao[1,2], Shan Yuan[2], Tianfu Han[2*], Wensheng Hou[1,2*]

GFP Bright Merged

35S::GFP

35S::GmFT2b-GFP

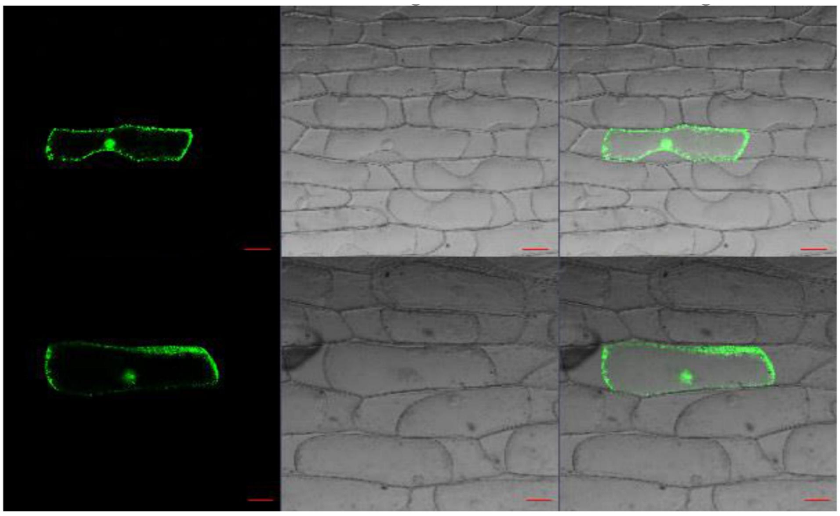

Figure S1. Subcellular localization of *GmFT2b*. The subcellular localization of the GmFT2b-GFP fusion protein was determined by transient expression in onion epidermal cells. The *35S:GFP* construct was the positive control.

```
GmFT2a    MPSGSRDPLVVGGVIGDVLDPFEYSIPMRVTYNNRDVSNGCEFKPSQVVNQPRVNIGGDD     60
GmFT2b    MPRGSRDPLVVGRVIGDVLDPFECSIPMRVTYNNKDVSNGCEFKPSQVVNQPRINIGGDD     60
Consensusmp gsrdplvvg vigdvldpfe sipmrvtynn dvsngcefkpsqvvnqpr niggdd

GmFT2a    LRNFYTLIAVDPDAPSPSDPNLREYLHWLVTDIPATTGASFGHEVVTYESPRPMMGIHRL    120
GmFT2b    FRNFYTLIAVDPDAPSPSDPNFREYLHWLVTDIPATTGPTFGHEVVTYENPRPMMGIHRI    120
Consensus rnfytliavdpdapspsdpn reylhwlvtdipattg  fghevvtye prpmmgihr

GmFT2a    VFVLFRQLGRETVYAPGWRQNFNTKEFAELYNLGLPVAAVYFNIQRESGSGGRRLY        176
GmFT2b    VFVLFRQQGRETVYAPGWRQNFITREFAELYNLGLPVAAVYFNIQRESGCGGRRLC        176
Consensusvfvlfrq gretvyapgwrqnf t efaelynlglpvaavyfniqresg ggrrl
```

Figure S2. Amino acid sequences alignments of *GmFT2a* and *GmFT2b* proteins. *GmFT2b* shares 90.91% amino acid sequence identity with its paralog *GmFT2a*.

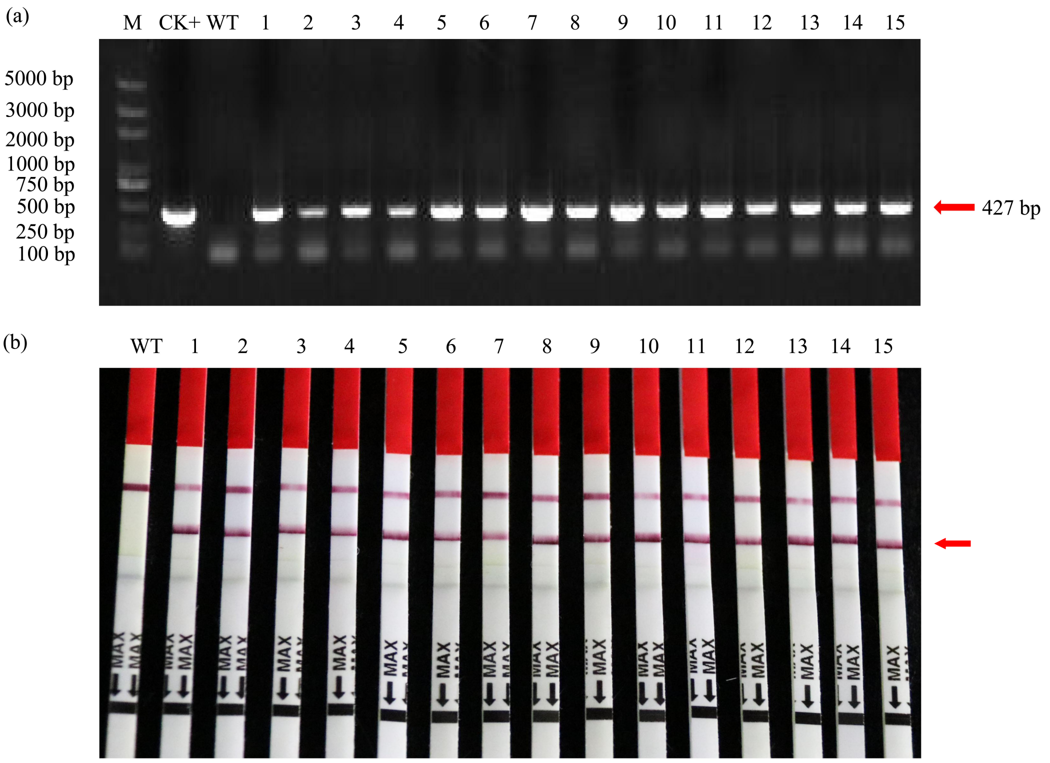

Figure S3. Detection of transgenic plants. (a) PCR analysis of genomic DNA of T2 transgenic plants using *bar* gene primers. The length of PCR product is 427 bp. M: DL2000 Plus; CK+: plasmid DNA; WT: wild-type soybean plant; lane 1-5: the T2 transgenic plants from line 7; lane 6-10: the T2 transgenic plants from line 14; lane 11-15: the T2 transgenic plants from line 22. (b) Detection by strip. WT: wild-type soybean plant; Labels 1-15, T2 homozygous mutant lines. The bands at red arrowhead indicate that *bar* is positive.

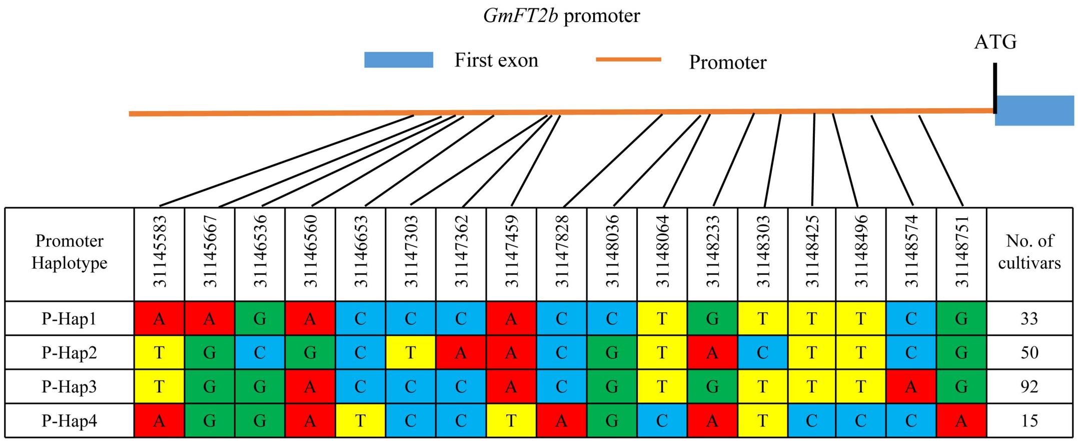

Promoter Haplotype	31145583	31145667	31146536	31146560	31146653	31147303	31147362	31147459	31147828	31148036	31148064	31148233	31148303	31148425	31148496	31148574	31148751	No. of cultivars
P-Hap1	A	A	G	A	C	C	C	A	C	C	T	G	T	T	T	C	G	33
P-Hap2	T	G	C	G	C	T	A	A	C	G	T	A	C	T	T	C	G	50
P-Hap3	T	G	G	A	C	C	C	A	C	G	T	G	T	T	T	A	G	92
P-Hap4	A	G	G	A	T	C	C	T	A	G	C	A	T	C	C	C	A	15

Figure S4. Analysis *of GmFT2b* promoter haplotype. About 3 kb *GmFT2b* promoter sequences were compared with those of Williams 82. Site numbering and physical positions are also based on the reference genome sequence of Williams 82. Four mainly haplotypes of *GmFT2b* promoter were listed. The nucleotides are highlighted in different colors. The number of cultivars carrying each haplotype are listed in the right column.

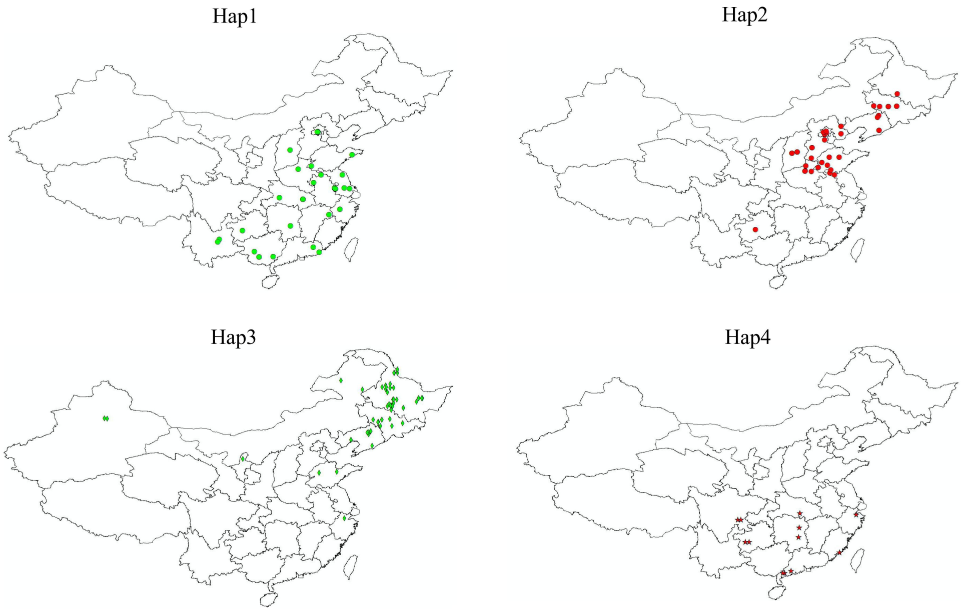

Figure S5. Geographic distribution of soybean accessions with major haplotypes of *GmFT2b*. The map is a map of China. Each dot represents a variety with the corresponding haplotype of *GmFT2b*.

Table S1 Primer sequences used in the present study

Primer name	Primer sequence (5'-3')	Gene ID
GmFT2b-CDS-F	ATGCCTCGTGGAAGTAGGGAC	Glyma.16g151000
GmFT2b-CDS-R	TTAACATAGCCTTCTTCCACC	
qGmFT1a-F	CCCAGCAAGGTAATCTGCAA	Glyma.18g298900
qGmFT1a-R	ATCGATTATTTCCTCATACGTACA	
qGmFT2a-F	ATGCACCTAGCCCAAGTGAC	Glyma.16g150700
qGmFT2a-R	TACACGGTCTCCCTACCCAG	
qGmFT3a-F	AGCGCCCAAATAAGAAAGGT	Glyma.16g044200
qGmFT3a-R	TGGTACATATGAGTGCTTCGGT	
qGmFT4-F	GGCAACCACTGGAGAAGAGA	Glyma.08g363100
qGmFT4-R	TGGAGCATGCACAATTTGTCT	
qGmFT5a-F	CACGGGAGAACCCTCTTGTTAT	Glyma.16g044100
qGmFT5a-R	GGTCTTCACCACCAACAGTAACC	
qGmAP1a-F	TGAACATGGGTGGCAATTAC	Glyma.16g091300
qGmAP1a-R	TGTCAAATGCCATACCAAAG	
qGmAP1b-F	TGGGAGCAGCCAAACTACAG	Glyma.01g064200
qGmAP1b-R	TGGTGCAGCTTCCTGATTGT	
qGmAP1c-F	GAAAGAAAAGGTTGCAGCTTC	Glyma.08g269800
qGmAP1c-R	GCATCCAAGGTGACAGGAAT	
qGmSOC1a-F	CGAGTTGCTTTTTTTCCCTAG	Glyma.18g224500
qGmSOC1a-R	TGAGTCTTTCCTCTCACCAT	
qGmSOC1b-F	AAGAAGCCCAACTGCAATGT	Glyma.09g266200
qGmSOC1b-R	GGGCTTCAGAAATGAGGAAAGG	
qGmFULa-F	CTCCCACAACAACACTAGCTC	Glyma.06g205800
qGmFULa-R	CCTACAAGACAATTCCAACACGA	
qGmLFY2-F	TGACGAAGGAAACATTAACACTGG	Glyma.06g163600
qGmLFY2-R	GCCTGAACCTGCATCAAGAA	
qGmAG-F	CAACAACCAGCTTCTTCGAGC	Glyma.15g088600
qGmAG-R	AGAAGGCAAACTACAAGGTAGC	
qGmActin-F	CGGTGGTTCTATCTTGGCATC	Glyma.18g52780
qGmActin-R	GTCTTTCGCTTCAATAACCCTA	
